# Factors Associated with Glycaemic Control among Diabetic Patients Managed at an Urban Hospital in Hanoi, Vietnam

**DOI:** 10.1155/2021/8886904

**Published:** 2021-02-24

**Authors:** Luu Quang Thuy, Hoang Thi Phuong Nam, Tran Thi Ha An, Bui Van San, Tran Nguyen Ngoc, Le Hong Trung, Pham Huy Tan, Nguyen Hoang Thanh

**Affiliations:** ^1^Viet Duc University Hospital, Hanoi 100000, Vietnam; ^2^National Geriatric Hospital, Hanoi 100000, Vietnam; ^3^National Institute of Mental Health-Bach Mai Hospital, Hanoi 100000, Vietnam; ^4^Hanoi Medical University, Hanoi 100000, Vietnam; ^5^Vinh Phuc General Hospital, Vinh Phuc 100000, Vietnam; ^6^Hanoi Medical University Hospital, Hanoi 100000, Vietnam

## Abstract

Type 2 diabetes (T2DM) epidemic is rising in Vietnam. Identifying associated factors with glycaemic control in patients with T2DM is vital to improve treatment outcomes. This study is aimed at examining the uncontrolled glycaemic level of patients with type 2 diabetes (T2DM) at an urban hospital in Hanoi, Vietnam, and determining associated factors. An observational longitudinal cohort survey was performed among T2DM patients. Glycaemic control was evaluated by using the HbA1c level ≥ 6.5% or fasting blood glucose level ≥ 7.5 g/mmol. Information about sociodemographic, clinical, and behavioral characteristics was collected. Multivariate mixed-effects logistic regression was employed to identify associated factors with control glycaemic level conditions. Among 189 T2DM patients, 70.4% had an uncontrolled glycaemic level. A higher number of comorbidities were associated with a lower likelihood of having uncontrolled glycaemic levels (OR = 0.71, *p* < 0.001, 95%CI = 0.52 − 0.98). Meanwhile, a higher body mass index (OR = 1.15, *p* < 0.05, 95%CI = 1.02 − 1.29), higher initial HbA1C (OR = 3.75, *p* < 0.01, 95%CI = 2.59 − 5.44), and higher initial fasting blood glucose levels (OR = 1.57, *p* < 0.01, 95%CI = 1.29 − 1.90) were positively associated with a higher risk of uncontrolled glycaemic levels. This study reveals that poor glycaemic control was common among T2DM patients in the urban hospital in Vietnam. Findings underlined the need for appropriate management strategies to control glycaemic levels and weight in this population.

## 1. Introduction

Diabetes mellitus (DM) is a global threat, given its significant health and social burden on people living with this condition [1]. The rate of diabetes among adult people elevated from 6.4% in 2010 to 8.3% in 2019, with more than 463 million people suffering T2DM around the world, according to a recent estimate [2]. This number is expected to reach approximately 578.4 million in 2030 and 630 million in 2045 [2].

Among patients with T2DM, controlling glycaemic levels is a prerequisite to prevent severe complications such as cardiovascular diseases, retinopathy diseases, or kidney diseases [2]. Many studies suggested that the prevalence of uncontrolled T2DM was substantially high. For example, a study in Saudi Arabia found that 43.1% of patients had uncontrolled T2DM [3]. Other studies in Ethiopia indicated that 59.2-70.8% of patients did not achieve controlled blood glucose levels [4–6]. This phenomenon was also observed in other countries such as India (78.2% among patients with diabetes complications) [7], Malaysia (79.7%) [8], and Brazil (76%) [9]. Factors affecting glycaemic control in patients with DM are complex and varied [10], including socioeconomic (e.g., advancing age, male, or low education), clinical (e.g., long duration of treatment, adherence to regular follow-up, having comorbidities, or type of medications), or behavioral (e.g., lack of physical activity or smoking) characteristics [3–9]. Understanding uncontrolled DM and factors associated with this condition is vital to inform strategies to enhance treatment outcomes.

In Vietnam, DM has still considered an epidemic, with approximately 5.5% of adults having diabetes in 2017 [11–13]. Several studies attempted to measure glycaemic control among T2DM patients in Vietnam. The first evidence in Diabcare-Asia 1998 study showed that only 18% of patients had optimal glycaemic levels [14]. Yokokawa et al. in Ho Chi Minh city suggested that the rate of poor blood glucose control ranged from 33% to 39% [15]. Another study in 43 hospitals of Vietnam revealed that 63.9% of T2DM patients had HbA1c ≥ 7.0% [16]. However, both studies were cross-sectional surveys and none of them established the associated factors with poor glycaemic control. This paper is aimed at examining the uncontrolled glycaemic level of patients with T2DM at an urban hospital in Hanoi, Vietnam, and determining associated factors.

## 2. Materials and Methods

### 2.1. Study Design

An observational retrospective longitudinal cohort survey was performed in October 2019 among T2DM patients at an urban hospital in Hanoi, Vietnam. In this study, we recruited T2DM patients who met the following inclusion criteria: (1) being diagnosed with T2DM regarding the Vietnam Ministry of Health's guideline [17]; (2) receiving T2DM treatment and management in the selected hospital at least one year (from October 2018 to October 2019); and (3) visiting the hospital during the study period. Patients (1) having cognitive disabilities, (2) being T2DM inpatients or outpatients without registration to the T2DM management program in the hospital, or (3) declining to be enrolled in the study were excluded.

The sample size was calculated by using the formula to estimate the proportion with specified absolute precision. With the confidence level = 95%, the expected proportion of uncontrolled glycaemic level = 79.7% (according to a previous study in Malaysia [8]), and the absolute precision = 0.06, the essential sample size was 173 patients. An addition of 15% of the total sample size was added to prevent people who did not accept to participate or not complete the interview, leading to a total of approximately 200 patients included in the sample frame. There were 189 patients (response rate of 94.5%) who agreed to be enrolled in the survey among 200 patients who were invited.

### 2.2. Data Collection Procedure

Patients were approached by the research team when they visited outpatient clinics for regular examination. Initially, they evaluated the eligible criteria by their physicians. After that, when they finished all procedures (e.g., examining medical conditions, having a blood test, and receiving drug prescription), and waited for medication dispense, they were invited to a private room to ensure their privacy. They were introduced briefly to the purpose of this study and the benefits of the participation by the research team and asked to sign a written informed consent if they agreed to participate in the study. Patients were then interviewed by undergraduate medical students of the Hanoi Medical University, who were trained carefully to have the necessary communication skills with patients. The interview was conducted according to the interview guideline. Finally, patients' clinical data from October 2018 to October 2019 were extracted from the electronic medical record system of the hospital.

### 2.3. Instruments

Our instruments included two parts: a structured questionnaire and a medical record. We developed a structured questionnaire to evaluate patients' characteristics including education, living area, and health behaviors. The questionnaire was piloted in five patients to test its feasibility and acceptability regarding the language, text, and content of the questions. Meanwhile, the medical record was used to extract clinical data comprising age, gender, comorbidities, the medication used, weight, height, body mass index, HbA1c level, and fasting blood glucose.

*Primary Outcome*: Glycaemic control was the primary outcome of this study. Patients were defined into the “uncontrolled” group if their HbA1c level was ≥6.5% and/or fasting blood glucose level was ≥7.5 mmol/L (according to the guideline of the Ministry of Health [17]); otherwise, they were classified into the “controlled” group. Information about patients' HbA1c level and fasting blood glucose level from October 2018 to October 2019 was extracted from the electronic medical record system. HbA1c and fasting blood glucose were measured according to the biochemical testing standard procedure of the hospital. Since the HbA1c and fasting blood glucose tests were not regular tests, the number of times that our sample took the tests ranged from one to four, and the last test was conducted on the date of data collection.

*Demographic Characteristics*: Age, gender (male/female), education (under high school/high school/above high school), and living location (rural/urban).

*Clinical Characteristics*: duration of diabetes (years), weight (kg), height (cm), and body mass index (kg/m^2^); name and number of comorbidities; types and the number of medications used (oral antidiabetics/insulin-injected/both), having hypertension (yes/no), and having dyslipidemia (yes/no). Regarding comorbidities, we recorded all diseases that patients suffered concurrently with T2DM such as cardiovascular diseases, retinopathy, neuropathy, and lung diseases.

*Behaviors*: Seven items of the Condition-specific Recommendations and Adherence scale were used to measure the adherence of patients to different recommended health behaviors for T2DM patients [18]. These behaviors included (1) taking prescription drugs daily; (2) checking blood glucose; (3) having a diet of diabetic patients; (4) stopping/reducing smoking; (5) stopping/reducing drinking alcohol; (6) checking for small wounds in the foot; and (7) exercise regularly. Each behavior has six levels of response from 0 “None of the time” to 5 “All of the time.” For “taking prescription drugs daily,” people having optimal adherence was those answering “all of the time,” while for other behaviors, optimal adherence was identified when patients responded “most of the time” or “all of the time” [18].

### 2.4. Statistical Analysis

Stata software version 14.0 was used to analyze the data. Descriptive statistics and multivariable regression models were applied in this study. Chi-squared and Mann-Whitney tests (due to nonnormal distribution of continuous variables) were performed to detect the differences in demographic, clinical, and behavioral characteristics between uncontrolled and controlled groups. A multivariate mixed-effects logistic regression was employed to identify associated factors with controlled glycaemic level overtime. This model can account for the subject-specific random intercepts and slopes as well as within-subject correlations. In this model, the outcome was controlled glycaemic level (controlled/uncontrolled), while independent variables included demographic characteristics (age, gender, education, living location), clinical characteristics (body mass index, duration of diabetes, number of comorbidities, number of medications used, initial HbA1C, and initial fasting blood glucose level), and behaviors (seven behaviors recommended for diabetes patients). These independent variables were selected based on previous literature in both Vietnam and global context, where revealed their significant associations with glycaemic control in T2DM patients [3–10]. Variance inflation factor was examined, and the result found that no collinearity existed. The level of statistical significance was set at a *p* value < 0.05.

### 2.5. Ethical Considerations

After patients were invited to be enrolled in the study, they were introduced to the study purposes and asked to join the study voluntarily. They were also informed that they could stop the interview at any time, and their withdrawal did not influence their treatment. If they agreed, they were asked to provide consent to the data collectors. This study was approved by the institutional review board of the hospital.

## 3. Results

Among 189 T2DM patients, 70.4% had uncontrolled glycaemic levels according to the last HbA1c and/or fasting blood glucose test. The mean age of the sample was 62.4 years (SD = 7.6), the mean age of the uncontrolled sample was 61.9 years (SD = 7.7), and the mean age of the controlled sample was 63.5 years (SD = 7.5) (*p* > 0.05). The majority of patients were female (55.6%) and had a high school degree or above (51.4%). Only the HbA1c and fasting blood glucose levels were found to be different between controlled and uncontrolled glycaemic level groups (*p* < 0.05). None of the other characteristics (e.g., diabetic medication, behaviors, comorbidities, medications, and body mass index) showed the differences between both groups (Table 1).

Table 2 indicates the results of the mixed-effects model. A higher number of comorbidities were found to be associated with a lower likelihood of having uncontrolled glycaemic levels (OR = 0.71, *p* < 0.001, 95%CI = 0.52 − 0.98). Meanwhile, a higher body mass index (OR = 1.15, *p* < 0.05, 95%CI = 1.02 − 1.29), a higher initial HbA1C (OR = 3.75, *p* < 0.01, 95%CI = 2.59 − 5.44), and a higher initial fasting blood glucose levels (OR = 1.57, *p* < 0.01, 95%CI = 1.29 − 1.90) were positively associated with a higher risk of uncontrolled glycaemic levels.

## 4. Discussion

Our current study found a high proportion of patients had poor control of blood glucose levels. This result was significantly higher than other studies in Saudi Arabia (43.1%) [3], but similar to some countries such as Ethiopia (59.2%-70.8%) [4–6], India (78.2%) [7], Malaysia (79.7%) [8], or Brazil (76%) [9]. Our result was high because we used both HbA1c and fasting blood glucose criteria to evaluate the glycaemic control. Thus, our assessment was more comprehensive than that in Ethiopia (59.2%), where the authors used only fasting blood glucose for evaluation [4], or in Saudi Arabia (43.1%) [3], where authors used merely HbA1c for evaluating blood glucose control. Other studies that had similar results to our study also used HbA1c or both criteria for evaluation. Therefore, our results are comparable with other studies. On the other hand, the results of this study might have a minor difference with other studies, which might be due to the variance of some factors such as age, comorbidities, or duration of diabetes [19, 20].

When comparing to previous studies in Vietnam, our finding was also somewhat similar to a national cross-sectional study in Vietnam with 36.1% patients having HbA1c < 7.0% [16] and better than results of DiabCare Asia 1998 study (18% patients having HbA1c < 7.0%) [14]. However, the rate of poor glycaemic control in our study was still at a high level. Several reasons can be used to explain this phenomenon. First, we observed a low frequency of blood glucose and HbA1c testing compared to the recommendations [17]. The highest number of blood glucose and HbA1c testing in our sample in 12 months was four. Moreover, we found the majority of patients did not adhere to checking blood glucose at home. These issues might cause difficulties in monitoring the progress of diabetes treatment. Second, more than a fifth of our sample did not follow the recommended behaviors such as reducing alcohol, eating a diabetic diet, or doing regular physical exercises. These reasons could greatly contribute to the substantially high rate of uncontrolled glycaemic level in our sample.

This study showed that body mass index was related to the ability to control blood sugar. Our research was similar to previous studies which showed that overweight and obese people were at a significantly higher risk of poor glycaemic control than those having average weight [19–21]. That was because being overweight and obese increased the risk of insulin resistance and further reduced insulin secretion as a function of the pancreas [19–21]. However, a previous study in Vietnam suggested that waist circumference measure was more informative than body mass index to evaluate the risk of noncommunicable diseases such as cardiovascular diseases or elevated blood glucose [22]. We suggested that further studies should employ this measure to gain a better assessment between body fat and diabetes control. Notably, our finding indicated that a higher number of comorbidities were related to a lower risk of poor glycaemic control. Previous studies showed that comorbidities were often related to uncontrolled blood glucose [7]. Our contradictory result might be explained that having several diseases facilitated patients having good diabetic medication adherence, which helped them to control diabetes and focus on treating other diseases. Apart from body mass index and comorbidities, we found no association between other factors and uncontrolled blood glucose levels. This result is different from some studies in the world where age, gender, education, location, duration of diabetes, or behavioral factors such as alcohol consumption, smoking, and physical activity were found to be associated with blood glucose control [3–9]. We supposed that the heterogeneity of our sample led to the insignificant associations between these factors and diabetes control.

The limitations of the study were that we only captured changes in blood sugar levels in one year because data were not available in the system. On the other hand, other variables such as clinical and behavioral indicators were collected only through a cross-sectional survey, which limited our ability to draw causality in this study. This study also had the limitation of using a small sample size with a convenient sampling method; thus, the possibility of extrapolation to other populations was limited. Finally, we used a generic instrument to measure recommended behaviors instead of specific measures for different behaviors (such as the International Physical Activity Questionnaire for physical activity, Morisky-8 for medication adherence, etc.), which might reduce our capacity to capture the actual level of these behaviors. Thus, further studies should be performed to address these knowledge gaps.

## 5. Conclusion

This study reveals that poor glycaemic control was common among T2DM patients in the urban hospital in Vietnam. Body mass index and comorbidities were significant associated factors with diabetes control. Findings underlined the need for appropriate management strategies to control glycaemic levels and weight in this population. Physicians should guide and encourage patients to monitor their blood glucose frequently at home, which helps them to be aware of their current blood glucose level and perform the necessary treatment to control it. Educational counseling services should be regularly provided to T2DM patients to promote healthy lifestyles to keep fit and reduce blood glucose, which, in turn, help to enhance diabetes management.

## Figures and Tables

**Table 1:** Sociodemographic, behavioral, and clinical characteristics.

Characteristics	Total	Controlled glycaemic level	Uncontrolled glycaemic level	*p*
Total, *n* (%)	189 (100.0)	56 (29.6)	133 (70.4)	
Demographic characteristics				
Gender, *n* (%)				
Female	105 (55.6)	37 (66.1)	68 (51.1)	0.06^∗^
Male	84 (44.4)	19 (33.9)	65 (48.9)	
Living location, *n* (%)				
Urban	164 (86.8)	49 (87.5)	115 (86.5)	0.85^∗^
Rural	25 (13.2)	7 (12.5)	18 (13.5)	
Education, *n* (%)				
Under high school	92 (48.7)	31 (55.4)	61 (45.9)	0.21^∗^
High school	30 (15.9)	5 (8.9)	25 (18.8)	
Above high school	67 (35.5)	20 (35.7)	47 (35.3)	
Age, mean ± SD	62.4 ± 7.6	63.5 ± 7.5	61.9 ± 7.7	0.07^∗∗^
Clinical characteristics				
Hypertension, *n* (%)	70 (37.0)	22 (39.3)	48 (36.1)	0.68^∗^
Dyslipidemia, *n* (%)	79 (41.8)	26 (46.4)	53 (39.9)	0.40^∗^
Diabetic medication, *n* (%)				
Oral antidiabetics	166 (90.2)	41 (94.4)	115 (88.5)	0.36^∗^
Insulin-injected	8 (4.4)	2 (3.7)	6 (4.6)	
Both	10 (5.4)	1 (1.9)	9 (6.9)	
Duration of diabetes (years), mean ± SD	7.9 ± 6.4	7.6 ± 6.3	8.0 ± 6.5	0.52^∗∗^
Number of comorbidities, mean ± SD	2.4 ± 1.4	2.6 ± 1.5	2.3 ± 1.4	0.18^∗∗^
Number of medications, mean ± SD	1.9 ± 1.4	2.0 ± 1.4	1.9 ± 1.4	0.90^∗∗^
Body mass index (kg/m^2^), mean ± SD	23.5 ± 3.0	22.8 ± 2.7	23.8 ± 3.1	0.09^∗∗^
Initial HbA1C (%), mean ± SD	6.7 ± 1.2	6.1 ± 0.7	7.0 ± 1.3	<0.01^∗∗^
Last HbA1C (%), mean ± SD	7.0 ± 2.1	5.7 ± 0.8	7.6 ± 2.3	<0.01^∗∗^
Initial fasting blood glucose level (mmol/L), mean ± SD	7.0 ± 2.0	6.2 ± 0.6	7.4 ± 2.3	<0.01^∗∗^
Last fasting blood glucose level (mmol/L), mean ± SD	7.3 ± 2.3	6.0 ± 0.8	7.8 ± 2.5	<0.01^∗∗^
Behavioral characteristics				
Adherence to behavioral recommendations				
Take prescription drugs daily, *n* (%)	173 (91.5)	53 (94.6)	120 (90.2)	0.32^∗^
Checking blood glucose, *n* (%)	36 (19.1)	6 (10.7)	30 (22.6)	0.06^∗^
Having diet of diabetic patients, *n* (%)	135 (71.4)	41 (73.2)	94 (70.7)	0.72^∗^
Stopping/reducing drinking alcohol, *n* (%)	150 (79.4)	46 (82.1)	104 (78.2)	0.54^∗^
Stopping/reducing smoking, *n* (%)	161 (85.2)	49 (87.5)	112 (84.2)	0.56^∗^
Checking for small wounds in the foot, *n* (%)	45 (23.8)	11 (19.6)	34 (25.6)	0.38^∗^
Exercising regularly, *n* (%)	135 (71.4)	37 (66.1)	98 (73.7)	0.29^∗^

^∗^Chi-squared test; ^∗∗^Mann-Whitney test.

**Table 2:** Associated factors with uncontrolled glycaemic levels.

Characteristics	OR	SE	*z*	95% CI		*p* value
Demographic characteristics						
Age (per year)	0.97	0.03	-1.04	0.92	1.02	0.30
Gender (female compared to male^a^)	0.48	0.19	-1.81	0.22	1.06	0.07
Living location (rural compared to urban^a^)	1.53	0.78	0.83	0.56	4.15	0.40
Education (compared to under high school^a^)						
High school	2.89	1.80	1.71	0.85	9.79	0.09
Above high school	0.74	0.31	-0.72	0.32	1.68	0.47
Clinical characteristics						
Diabetic medication (compared to oral antidiabetics^a^)						
Insulin-injected	0.47	0.47	-0.75	0.06	3.38	0.45
Both	3.88	4.99	1.06	0.31	48.09	0.29
Duration of diabetes (per year)	1.01	0.03	0.22	0.94	1.08	0.82
Number of comorbidities (per disease)	0.71	0.12	-2.09	0.52	0.98	**0.04**
Number of medications used (per drug)	1.26	0.17	1.70	0.97	1.64	0.09
Body mass index (per 1 kg/m^2^)	1.15	0.07	2.25	1.02	1.29	**0.03**
Initial HbA1C (per %)	3.75	0.71	6.97	2.59	5.44	**<0.01**
Initial fasting blood glucose level (per 1 mmol/L)	1.57	0.16	4.51	1.29	1.90	**<0.01**
Hypertension (yes compared to no)	0.66	0.28	-0.98	0.28	1.52	0.33
Dyslipidemia (yes compared to no)	0.70	0.27	-0.92	0.33	1.50	0.36
Behavioral characteristics						
Take prescription drugs daily (yes compared to no)	0.58	0.45	-0.70	0.13	2.64	0.48
Checking blood glucose (adhere compared to no adhere)	1.75	0.95	1.04	0.61	5.05	0.30
Having diet of diabetic patients (adhere compared to no adhere)	0.91	0.35	-0.25	0.42	1.95	0.80
Stopping/reducing drinking alcohol (adhere compared to no adhere)	1.00	0.56	0.01	0.34	2.99	0.99
Stopping/reducing smoking (adhere compared to no adhere)	1.01	0.62	0.01	0.30	3.39	0.99
Checking for small wounds in the foot (adhere compared to no adhere)	2.45	1.18	1.86	0.95	6.31	0.06
Exercising regularly (adhere compared to no adhere)	1.22	0.50	0.50	0.55	2.71	0.62

*^a^*Reference group.

## Data Availability

The data used to support the findings of this study are available from the corresponding author upon request.
